# (2*E*,2′*E*)-Dimethyl 2,2′-[(phenyl­aza­nedi­yl)bis­(methyl­ene)]bis­(3-phenyl­acrylate)

**DOI:** 10.1107/S1600536812029042

**Published:** 2012-06-30

**Authors:** V. Sabari, R. Selvakumar, M. Bakthadoss, S. Aravindhan

**Affiliations:** a Department of Physics, Presidency College, Chennai 600 005, India; b Department of Organic Chemistry, University of Madras, Chennai 600 025, India; c Department of Organic Chemistry, University of mMdras, Chennai 600 025, India

## Abstract

The C=C double bonds in the title compound, C_28_H_27_NO_4_, adopt an *E* conformation. In the crystal, pairs of C—H⋯O hydrogen bonds link the mol­ecules into inversion dimers.

## Related literature

For applications of acrylate derivatives, see: De Fraine & Martin (1991 ▶). For resonance effects of the acrylate moiety, see: Merlino (1971 ▶); Varghese *et al.* (1986 ▶).
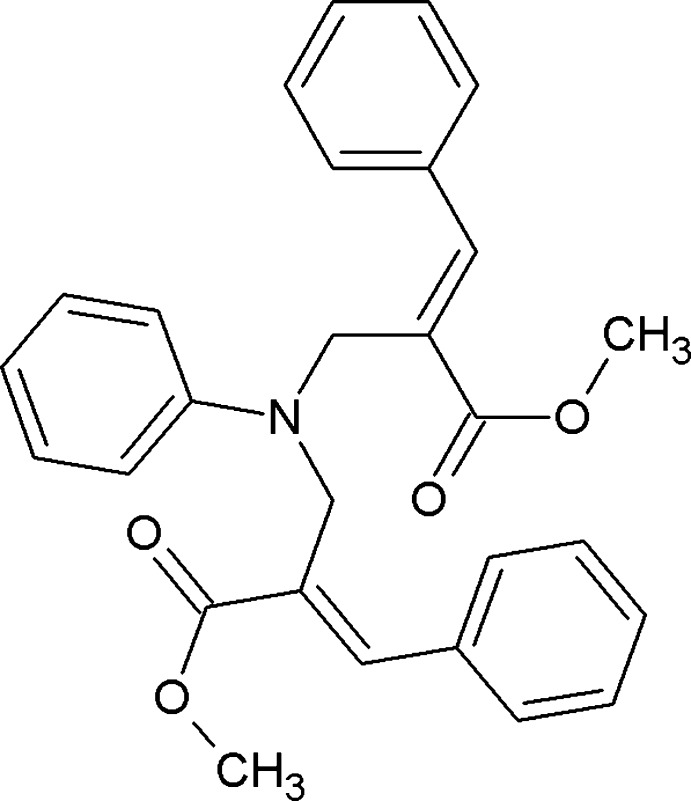


## Experimental

### 

#### Crystal data

C_28_H_27_NO_4_
*M* *_r_* = 441.51
Triclinic, 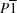

*a* = 9.9099 (2) Å
*b* = 11.7327 (2) Å
*c* = 12.4079 (4) Å
α = 101.131 (2)°
β = 106.039 (2)°
γ = 114.817 (1)°
*V* = 1176.86 (5) Å^3^
*Z* = 2
Mo *K*α radiation
μ = 0.08 mm^−1^
*T* = 298 K
0.32 × 0.20 × 0.10 mm

#### Data collection

Bruker APEXII CCD area-detector diffractometer
Absorption correction: multi-scan (*SADABS*; Bruker, 2008 ▶) *T*_min_ = 0.972, *T*_max_ = 0.992
15565 measured reflections
5710 independent reflections
3683 reflections with *I* > 2σ(*I*)
*R*_int_ = 0.024

#### Refinement

*R*[*F* ^2^ > 2σ(*F* ^2^)] = 0.047
*wR*(*F* ^2^) = 0.127
*S* = 1.03
5710 reflections
300 parameters
H-atom parameters constrained
Δρ_max_ = 0.26 e Å^−3^
Δρ_min_ = −0.19 e Å^−3^

### 

Data collection: *APEX2* (Bruker, 2008 ▶); cell refinement: *SAINT* (Bruker, 2008 ▶); data reduction: *SAINT*; program(s) used to solve structure: *SHELXS97* (Sheldrick, 2008 ▶); program(s) used to refine structure: *SHELXL97* (Sheldrick, 2008 ▶); molecular graphics: *ORTEP-3 for Windows* (Farrugia, 1997 ▶); software used to prepare material for publication: *PLATON* (Spek, 2009 ▶).

## Supplementary Material

Crystal structure: contains datablock(s) I, global. DOI: 10.1107/S1600536812029042/bt5946sup1.cif

Structure factors: contains datablock(s) I. DOI: 10.1107/S1600536812029042/bt5946Isup2.hkl

Supplementary material file. DOI: 10.1107/S1600536812029042/bt5946Isup3.cml

Additional supplementary materials:  crystallographic information; 3D view; checkCIF report

## Figures and Tables

**Table 1:** Hydrogen-bond geometry (Å, °)

*D*—H⋯*A*	*D*—H	H⋯*A*	*D*⋯*A*	*D*—H⋯*A*
C9—H9*A*⋯O1^i^	0.97	2.52	3.474 (2)	167

Symmetry code: (i) 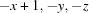.

**Fig. 1.**
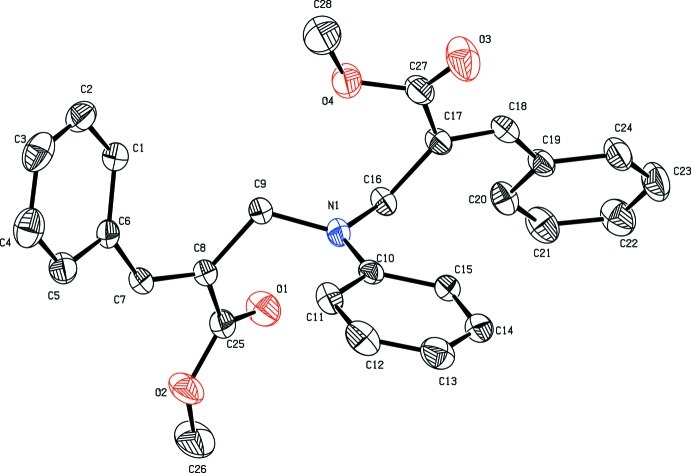
The molecular structure of the title compound, showing 30% probability displacement ellipsoids for non-H atoms.

**Fig. 2.**
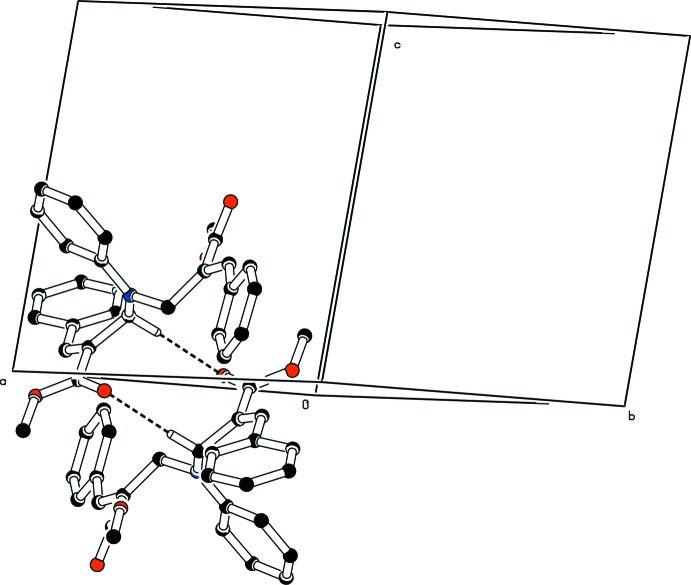
A view of the crystal packing. H atoms not involved in hydrogen bonding (dashed lines) have been omitted for clarity.

## supplementary crystallographic information

### Comment

Acrylate and their derivatives are important compounds because of their agrochemical and medical applications (De Fraine & Martin, 1991). In view of this medicinal importance, the crystal structure determination of the title compound was carried out and the results are presented here.

Fig. 1.shows a displacement ellipsoid plot of the title compound with the atom numbering scheme. The molecule adopts an E configuration about the C7=C8 and C17=C18 double bonds. The dihedral angle between the two aromatic rings (C1—C6) and (C10—C15) is 70.40 (4)°, (C1—C6) and (C19—C24) is 61.55 (6) °. The significant difference in length of the C27—O4 = 1.325 (2) Å and C28—O4 = 1.437 (2) Å bonds is attributed to a partial contribution from the O^-^—C = O^+^—C resonance structure of the O3=C27—O4—C28 group and C25—O2= 1.337 (2) Å and C26—O2= 1.439 (2) Å bonds is attributed to a partial contribution from the O^-^—C = O^+^—C resonance structure of the O1=C25—O4—C26 group (Merlino, 1971). This feature, commonly observed in the carboxylic ester group of the substituents in various compounds gives average values of 1.340 Å and 1.447 Å respectively for these bonds (Varghese *et al.*, 1986).

The crystal packing is stabilized by intermolecular nonclassical C—H···O hydrogen bonds linking the molecules into centrosymmetric dimers. A packing view of the title compound is shown in Fig. 2.

### Experimental

A mixture of (*Z*)-methyl 2-(bromomethyl)-3-phenylacrylate (2 mmol) and aniline (1 mmol) in the presence of potassium carbonate (4 mmol) in dry acetonitrile (10 ml) was stirred at room temperature for 3 h. After the completion of the reaction as indicated by TLC, the reaction mixture was concentrated and the resulting crude mass was diluted with water (20 ml) and extracted with ethyl acetate (3 *x* 20 ml). The organic layer was washed with brine (2 *x* 20 ml) and dried over anhydrous sodium sulfate. The organic layer was concentrated, which successfully provide the crude final product ((2E,2'*E*)-dimethyl 2,2'-(phenylazanediyl)bis(methylene)bis(3-phenylacrylate)). The final product was purified by column chromatography on silica gel to afford the title compound in 35% yields.

### Refinement

The hydrogen atoms were placed in calculated positions with C—H = 0.93 Å to 0.97 Å and refined in the riding model with fixed isotropic displacement parameters: *U*_iso_(H) = 1.5U_eq_(C) for methyl group and *U*_iso_(H) = 1.2U_eq_(C) for other groups.

### Crystal data

**Table:**

C_28_H_27_NO_4_	*Z* = 2
*M**_r_* = 441.51	*F*(000) = 468
Triclinic, *P*1	*D*_x_ = 1.246 Mg m^−^^3^
Hall symbol: -P 1	Mo *K*α radiation, λ = 0.71073 Å
*a* = 9.9099 (2) Å	Cell parameters from 5710 reflections
*b* = 11.7327 (2) Å	θ = 1.8–28.5°
*c* = 12.4079 (4) Å	µ = 0.08 mm^−^^1^
α = 101.131 (2)°	*T* = 298 K
β = 106.039 (2)°	Triclinic, colourless
γ = 114.817 (1)°	0.32 × 0.20 × 0.10 mm
*V* = 1176.86 (5) Å^3^	

### Data collection

**Table:**

Bruker APEXII CCD area-detector diffractometer	5710 independent reflections
Radiation source: fine-focus sealed tube	3683 reflections with *I* > 2σ(*I*)
Graphite monochromator	*R*_int_ = 0.024
ω and φ scans	θ_max_ = 28.5°, θ_min_ = 1.8°
Absorption correction: multi-scan (*SADABS*; Bruker, 2008)	*h* = −12→13
*T*_min_ = 0.972, *T*_max_ = 0.992	*k* = −15→15
15565 measured reflections	*l* = −16→16

### Refinement

**Table:**

Refinement on *F*^2^	Primary atom site location: structure-invariant direct methods
Least-squares matrix: full	Secondary atom site location: difference Fourier map
*R*[*F*^2^ > 2σ(*F*^2^)] = 0.047	Hydrogen site location: inferred from neighbouring sites
*wR*(*F*^2^) = 0.127	H-atom parameters constrained
*S* = 1.03	*w* = 1/[σ^2^(*F*_o_^2^) + (0.0521*P*)^2^ + 0.1829*P*] where *P* = (*F*_o_^2^ + 2*F*_c_^2^)/3
5710 reflections	(Δ/σ)_max_ < 0.001
300 parameters	Δρ_max_ = 0.26 e Å^−^^3^
0 restraints	Δρ_min_ = −0.19 e Å^−^^3^

### Special details

**Table:**

Geometry. All e.s.d.'s (except the e.s.d. in the dihedral angle between two l.s. planes) are estimated using the full covariance matrix. The cell e.s.d.'s are taken into account individually in the estimation of e.s.d.'s in distances, angles and torsion angles; correlations between e.s.d.'s in cell parameters are only used when they are defined by crystal symmetry. An approximate (isotropic) treatment of cell e.s.d.'s is used for estimating e.s.d.'s involving l.s. planes.
Refinement. Refinement of *F*^2^ against ALL reflections. The weighted *R*-factor *wR* and goodness of fit *S* are based on *F*^2^, conventional *R*-factors *R* are based on *F*, with *F* set to zero for negative *F*^2^. The threshold expression of *F*^2^ > σ(*F*^2^) is used only for calculating *R*-factors(gt) *etc*. and is not relevant to the choice of reflections for refinement. *R*-factors based on *F*^2^ are statistically about twice as large as those based on *F*, and *R*- factors based on ALL data will be even larger.

### Fractional atomic coordinates and isotropic or equivalent isotropic displacement parameters (Å^2^)

**Table:**

	*x*	*y*	*z*	*U*_iso_*/*U*_eq_	
C1	0.41831 (18)	−0.27590 (16)	0.18680 (14)	0.0505 (4)	
H1	0.3986	−0.2081	0.1731	0.061*	
C2	0.3444 (2)	−0.34918 (18)	0.24884 (16)	0.0624 (5)	
H2	0.2763	−0.3297	0.2775	0.075*	
C3	0.3709 (2)	−0.4505 (2)	0.26837 (17)	0.0694 (5)	
H3	0.3219	−0.4987	0.3111	0.083*	
C4	0.4697 (2)	−0.48088 (19)	0.22485 (17)	0.0664 (5)	
H4	0.4866	−0.5503	0.2373	0.080*	
C5	0.54353 (19)	−0.40857 (16)	0.16293 (15)	0.0525 (4)	
H5	0.6089	−0.4307	0.1327	0.063*	
C6	0.52232 (17)	−0.30277 (14)	0.14460 (13)	0.0418 (3)	
C7	0.60328 (17)	−0.23200 (14)	0.07556 (12)	0.0427 (3)	
H7	0.6177	−0.2840	0.0180	0.051*	
C8	0.65925 (17)	−0.10413 (14)	0.08314 (12)	0.0402 (3)	
C9	0.65417 (17)	0.00032 (14)	0.17295 (13)	0.0427 (3)	
H9A	0.5591	0.0062	0.1343	0.051*	
H9B	0.6412	−0.0304	0.2385	0.051*	
C10	0.93968 (17)	0.16227 (14)	0.30881 (12)	0.0365 (3)	
C11	0.9588 (2)	0.06137 (16)	0.34304 (13)	0.0476 (4)	
H11	0.8730	−0.0267	0.3075	0.057*	
C12	1.1034 (2)	0.09086 (19)	0.42885 (14)	0.0598 (5)	
H12	1.1132	0.0222	0.4504	0.072*	
C13	1.2329 (2)	0.21927 (19)	0.48300 (15)	0.0627 (5)	
H13	1.3297	0.2382	0.5409	0.075*	
C14	1.2162 (2)	0.31928 (17)	0.44981 (15)	0.0557 (4)	
H14	1.3035	0.4067	0.4855	0.067*	
C15	1.07327 (17)	0.29301 (14)	0.36500 (13)	0.0435 (3)	
H15	1.0652	0.3629	0.3447	0.052*	
C16	0.77545 (19)	0.24172 (14)	0.19443 (13)	0.0424 (3)	
H16A	0.6728	0.2021	0.1264	0.051*	
H16B	0.8608	0.2908	0.1699	0.051*	
C17	0.77845 (18)	0.34132 (15)	0.29512 (13)	0.0426 (3)	
C18	0.83568 (18)	0.47221 (15)	0.31417 (13)	0.0460 (4)	
H18	0.8367	0.5199	0.3839	0.055*	
C19	0.89688 (18)	0.55354 (15)	0.24480 (13)	0.0440 (3)	
C20	0.8597 (2)	0.50340 (16)	0.12296 (14)	0.0512 (4)	
H20	0.7963	0.4112	0.0815	0.061*	
C21	0.9149 (2)	0.58743 (18)	0.06251 (16)	0.0619 (5)	
H21	0.8882	0.5517	−0.0191	0.074*	
C22	1.0091 (3)	0.72337 (19)	0.12183 (18)	0.0707 (5)	
H22	1.0481	0.7799	0.0812	0.085*	
C23	1.0455 (3)	0.77566 (18)	0.24174 (19)	0.0718 (5)	
H23	1.1089	0.8680	0.2822	0.086*	
C24	0.9889 (2)	0.69226 (16)	0.30240 (16)	0.0582 (4)	
H24	1.0124	0.7292	0.3832	0.070*	
C25	0.72072 (18)	−0.06039 (16)	−0.00643 (14)	0.0468 (4)	
C26	0.8187 (3)	−0.1112 (2)	−0.15181 (19)	0.0900 (7)	
H26A	0.7303	−0.1187	−0.2152	0.135*	
H26B	0.8486	−0.1746	−0.1821	0.135*	
H26C	0.9099	−0.0219	−0.1217	0.135*	
C27	0.7138 (2)	0.29464 (17)	0.38288 (15)	0.0530 (4)	
C28	0.5352 (3)	0.1088 (2)	0.4154 (2)	0.0999 (8)	
H28A	0.6222	0.1220	0.4839	0.150*	
H28B	0.4601	0.0144	0.3739	0.150*	
H28C	0.4801	0.1518	0.4414	0.150*	
N1	0.79578 (14)	0.13407 (11)	0.22255 (10)	0.0407 (3)	
O1	0.72348 (16)	0.03324 (13)	−0.03387 (11)	0.0649 (3)	
O2	0.76968 (17)	−0.13876 (12)	−0.05645 (11)	0.0697 (4)	
O3	0.7549 (2)	0.36236 (14)	0.48407 (12)	0.0899 (5)	
O4	0.59991 (17)	0.16605 (12)	0.33627 (12)	0.0769 (4)	

### Atomic displacement parameters (Å^2^)

**Table:**

	*U*^11^	*U*^22^	*U*^33^	*U*^12^	*U*^13^	*U*^23^
C1	0.0439 (8)	0.0400 (9)	0.0580 (10)	0.0154 (7)	0.0226 (8)	0.0087 (7)
C2	0.0484 (10)	0.0593 (12)	0.0636 (11)	0.0140 (9)	0.0287 (9)	0.0121 (9)
C3	0.0591 (11)	0.0653 (13)	0.0618 (11)	0.0110 (10)	0.0231 (10)	0.0281 (10)
C4	0.0644 (11)	0.0522 (11)	0.0699 (12)	0.0216 (10)	0.0174 (10)	0.0285 (10)
C5	0.0503 (9)	0.0434 (9)	0.0567 (10)	0.0222 (8)	0.0173 (8)	0.0130 (8)
C6	0.0388 (7)	0.0313 (8)	0.0409 (8)	0.0108 (6)	0.0131 (6)	0.0043 (6)
C7	0.0441 (8)	0.0353 (8)	0.0431 (8)	0.0181 (7)	0.0189 (7)	0.0046 (6)
C8	0.0384 (7)	0.0345 (8)	0.0407 (8)	0.0155 (7)	0.0157 (6)	0.0059 (6)
C9	0.0419 (8)	0.0320 (8)	0.0491 (8)	0.0155 (7)	0.0210 (7)	0.0076 (6)
C10	0.0439 (8)	0.0350 (8)	0.0350 (7)	0.0203 (7)	0.0225 (6)	0.0104 (6)
C11	0.0599 (10)	0.0387 (9)	0.0416 (8)	0.0237 (8)	0.0199 (8)	0.0123 (7)
C12	0.0810 (13)	0.0633 (12)	0.0442 (9)	0.0476 (11)	0.0199 (9)	0.0178 (9)
C13	0.0589 (11)	0.0724 (13)	0.0460 (9)	0.0387 (11)	0.0090 (8)	0.0040 (9)
C14	0.0464 (9)	0.0506 (10)	0.0548 (10)	0.0209 (8)	0.0175 (8)	0.0002 (8)
C15	0.0447 (8)	0.0366 (8)	0.0483 (8)	0.0190 (7)	0.0235 (7)	0.0097 (7)
C16	0.0516 (9)	0.0354 (8)	0.0420 (8)	0.0242 (7)	0.0194 (7)	0.0110 (6)
C17	0.0477 (8)	0.0388 (8)	0.0447 (8)	0.0252 (7)	0.0198 (7)	0.0110 (7)
C18	0.0547 (9)	0.0427 (9)	0.0458 (8)	0.0298 (8)	0.0226 (7)	0.0099 (7)
C19	0.0500 (8)	0.0371 (8)	0.0503 (9)	0.0279 (7)	0.0197 (7)	0.0124 (7)
C20	0.0635 (10)	0.0406 (9)	0.0483 (9)	0.0297 (8)	0.0166 (8)	0.0136 (7)
C21	0.0878 (13)	0.0560 (11)	0.0545 (10)	0.0443 (11)	0.0290 (10)	0.0243 (9)
C22	0.0986 (15)	0.0569 (12)	0.0821 (14)	0.0463 (12)	0.0493 (12)	0.0386 (11)
C23	0.0914 (14)	0.0364 (10)	0.0839 (14)	0.0281 (10)	0.0388 (12)	0.0177 (10)
C24	0.0734 (12)	0.0406 (9)	0.0602 (10)	0.0299 (9)	0.0293 (9)	0.0104 (8)
C25	0.0473 (9)	0.0390 (9)	0.0464 (8)	0.0175 (7)	0.0198 (7)	0.0081 (7)
C26	0.1282 (19)	0.0856 (16)	0.0804 (14)	0.0509 (15)	0.0781 (15)	0.0305 (12)
C27	0.0664 (11)	0.0444 (10)	0.0567 (10)	0.0303 (9)	0.0339 (9)	0.0156 (8)
C28	0.1206 (19)	0.0731 (15)	0.1195 (19)	0.0308 (14)	0.0928 (17)	0.0376 (14)
N1	0.0428 (7)	0.0290 (6)	0.0458 (7)	0.0167 (6)	0.0161 (6)	0.0100 (5)
O1	0.0853 (9)	0.0595 (8)	0.0673 (8)	0.0399 (7)	0.0417 (7)	0.0318 (7)
O2	0.1020 (10)	0.0596 (8)	0.0761 (8)	0.0452 (8)	0.0650 (8)	0.0272 (7)
O3	0.1303 (13)	0.0634 (9)	0.0627 (8)	0.0304 (9)	0.0559 (9)	0.0136 (7)
O4	0.0862 (9)	0.0504 (8)	0.0820 (9)	0.0144 (7)	0.0586 (8)	0.0099 (7)

### Geometric parameters (Å, º)

**Table:**

C1—C2	1.381 (2)	C16—N1	1.4499 (17)
C1—C6	1.394 (2)	C16—C17	1.5211 (19)
C1—H1	0.9300	C16—H16A	0.9700
C2—C3	1.370 (3)	C16—H16B	0.9700
C2—H2	0.9300	C17—C18	1.338 (2)
C3—C4	1.375 (3)	C17—C27	1.489 (2)
C3—H3	0.9300	C18—C19	1.462 (2)
C4—C5	1.373 (2)	C18—H18	0.9300
C4—H4	0.9300	C19—C20	1.391 (2)
C5—C6	1.392 (2)	C19—C24	1.391 (2)
C5—H5	0.9300	C20—C21	1.376 (2)
C6—C7	1.4704 (19)	C20—H20	0.9300
C7—C8	1.3362 (19)	C21—C22	1.369 (3)
C7—H7	0.9300	C21—H21	0.9300
C8—C25	1.484 (2)	C22—C23	1.373 (3)
C8—C9	1.5172 (19)	C22—H22	0.9300
C9—N1	1.4533 (18)	C23—C24	1.377 (2)
C9—H9A	0.9700	C23—H23	0.9300
C9—H9B	0.9700	C24—H24	0.9300
C10—N1	1.3830 (18)	C25—O1	1.2032 (18)
C10—C11	1.3985 (19)	C25—O2	1.3367 (18)
C10—C15	1.401 (2)	C26—O2	1.440 (2)
C11—C12	1.380 (2)	C26—H26A	0.9600
C11—H11	0.9300	C26—H26B	0.9600
C12—C13	1.370 (3)	C26—H26C	0.9600
C12—H12	0.9300	C27—O3	1.1976 (19)
C13—C14	1.373 (2)	C27—O4	1.326 (2)
C13—H13	0.9300	C28—O4	1.438 (2)
C14—C15	1.376 (2)	C28—H28A	0.9600
C14—H14	0.9300	C28—H28B	0.9600
C15—H15	0.9300	C28—H28C	0.9600
			
C2—C1—C6	120.52 (15)	N1—C16—H16B	108.3
C2—C1—H1	119.7	C17—C16—H16B	108.3
C6—C1—H1	119.7	H16A—C16—H16B	107.4
C3—C2—C1	120.32 (16)	C18—C17—C27	114.62 (13)
C3—C2—H2	119.8	C18—C17—C16	125.71 (13)
C1—C2—H2	119.8	C27—C17—C16	119.67 (13)
C2—C3—C4	120.09 (16)	C17—C18—C19	131.42 (13)
C2—C3—H3	120.0	C17—C18—H18	114.3
C4—C3—H3	120.0	C19—C18—H18	114.3
C5—C4—C3	119.93 (17)	C20—C19—C24	117.28 (15)
C5—C4—H4	120.0	C20—C19—C18	124.78 (14)
C3—C4—H4	120.0	C24—C19—C18	117.74 (14)
C4—C5—C6	121.21 (16)	C21—C20—C19	121.32 (15)
C4—C5—H5	119.4	C21—C20—H20	119.3
C6—C5—H5	119.4	C19—C20—H20	119.3
C5—C6—C1	117.88 (14)	C22—C21—C20	120.34 (16)
C5—C6—C7	117.79 (13)	C22—C21—H21	119.8
C1—C6—C7	124.22 (13)	C20—C21—H21	119.8
C8—C7—C6	129.70 (12)	C21—C22—C23	119.52 (17)
C8—C7—H7	115.1	C21—C22—H22	120.2
C6—C7—H7	115.1	C23—C22—H22	120.2
C7—C8—C25	119.20 (12)	C22—C23—C24	120.42 (17)
C7—C8—C9	124.47 (12)	C22—C23—H23	119.8
C25—C8—C9	116.18 (12)	C24—C23—H23	119.8
N1—C9—C8	115.32 (11)	C23—C24—C19	121.07 (16)
N1—C9—H9A	108.4	C23—C24—H24	119.5
C8—C9—H9A	108.4	C19—C24—H24	119.5
N1—C9—H9B	108.4	O1—C25—O2	123.05 (14)
C8—C9—H9B	108.4	O1—C25—C8	124.34 (13)
H9A—C9—H9B	107.5	O2—C25—C8	112.58 (13)
N1—C10—C11	121.47 (13)	O2—C26—H26A	109.5
N1—C10—C15	121.47 (12)	O2—C26—H26B	109.5
C11—C10—C15	117.06 (13)	H26A—C26—H26B	109.5
C12—C11—C10	120.84 (15)	O2—C26—H26C	109.5
C12—C11—H11	119.6	H26A—C26—H26C	109.5
C10—C11—H11	119.6	H26B—C26—H26C	109.5
C13—C12—C11	121.37 (16)	O3—C27—O4	121.91 (15)
C13—C12—H12	119.3	O3—C27—C17	125.83 (16)
C11—C12—H12	119.3	O4—C27—C17	112.25 (13)
C12—C13—C14	118.45 (16)	O4—C28—H28A	109.5
C12—C13—H13	120.8	O4—C28—H28B	109.5
C14—C13—H13	120.8	H28A—C28—H28B	109.5
C13—C14—C15	121.48 (16)	O4—C28—H28C	109.5
C13—C14—H14	119.3	H28A—C28—H28C	109.5
C15—C14—H14	119.3	H28B—C28—H28C	109.5
C14—C15—C10	120.80 (14)	C10—N1—C16	120.33 (11)
C14—C15—H15	119.6	C10—N1—C9	120.62 (11)
C10—C15—H15	119.6	C16—N1—C9	118.29 (11)
N1—C16—C17	115.91 (11)	C25—O2—C26	116.69 (14)
N1—C16—H16A	108.3	C27—O4—C28	116.80 (15)
C17—C16—H16A	108.3		
			
C6—C1—C2—C3	−0.8 (3)	C24—C19—C20—C21	1.5 (2)
C1—C2—C3—C4	−0.8 (3)	C18—C19—C20—C21	176.33 (15)
C2—C3—C4—C5	0.8 (3)	C19—C20—C21—C22	0.3 (3)
C3—C4—C5—C6	1.0 (3)	C20—C21—C22—C23	−1.3 (3)
C4—C5—C6—C1	−2.6 (2)	C21—C22—C23—C24	0.4 (3)
C4—C5—C6—C7	−178.95 (15)	C22—C23—C24—C19	1.5 (3)
C2—C1—C6—C5	2.5 (2)	C20—C19—C24—C23	−2.3 (2)
C2—C1—C6—C7	178.59 (15)	C18—C19—C24—C23	−177.57 (16)
C5—C6—C7—C8	−150.06 (16)	C7—C8—C25—O1	154.50 (16)
C1—C6—C7—C8	33.8 (2)	C9—C8—C25—O1	−21.3 (2)
C6—C7—C8—C25	−173.35 (14)	C7—C8—C25—O2	−23.9 (2)
C6—C7—C8—C9	2.1 (2)	C9—C8—C25—O2	160.27 (13)
C7—C8—C9—N1	141.60 (14)	C18—C17—C27—O3	−26.7 (2)
C25—C8—C9—N1	−42.83 (18)	C16—C17—C27—O3	153.25 (18)
N1—C10—C11—C12	179.68 (13)	C18—C17—C27—O4	152.40 (14)
C15—C10—C11—C12	0.3 (2)	C16—C17—C27—O4	−27.7 (2)
C10—C11—C12—C13	−0.2 (2)	C11—C10—N1—C16	175.89 (12)
C11—C12—C13—C14	−0.2 (2)	C15—C10—N1—C16	−4.70 (18)
C12—C13—C14—C15	0.5 (2)	C11—C10—N1—C9	6.04 (18)
C13—C14—C15—C10	−0.5 (2)	C15—C10—N1—C9	−174.56 (12)
N1—C10—C15—C14	−179.34 (12)	C17—C16—N1—C10	−64.45 (17)
C11—C10—C15—C14	0.09 (19)	C17—C16—N1—C9	105.64 (15)
N1—C16—C17—C18	147.18 (15)	C8—C9—N1—C10	−75.60 (16)
N1—C16—C17—C27	−32.7 (2)	C8—C9—N1—C16	114.34 (14)
C27—C17—C18—C19	−174.80 (15)	O1—C25—O2—C26	−3.5 (3)
C16—C17—C18—C19	5.3 (3)	C8—C25—O2—C26	174.95 (16)
C17—C18—C19—C20	22.5 (3)	O3—C27—O4—C28	−4.1 (3)
C17—C18—C19—C24	−162.63 (16)	C17—C27—O4—C28	176.82 (16)

### Hydrogen-bond geometry (Å, º)

**Table:**

*D*—H···*A*	*D*—H	H···*A*	*D*···*A*	*D*—H···*A*
C9—H9*A*···O1^i^	0.97	2.52	3.474 (2)	167

Symmetry code: (i) −*x*+1, −*y*, −*z*.
